# A scoping review of EFL learners’ interlanguage pragmatic development

**DOI:** 10.1371/journal.pone.0344811

**Published:** 2026-03-13

**Authors:** Huaqiang Li, Zalina Mohd Kasim, Lay Hoon Ang

**Affiliations:** 1 Department of English, Faculty of Modern Languages and Communication, Universiti Putra Malaysia, Serdang, Selangor, Malaysia; 2 School of Foreign Languages, Xinxiang University, Xinxiang, Henan, China; National University of Malaysia Faculty of Education: Universiti Kebangsaan Malaysia Fakulti Pendidikan, MALAYSIA

## Abstract

Interlanguage pragmatics (ILP) development refers to how L2 learners manage actions, identities, and social relations through language in interaction. The rapid transition to multimodal, digitally mediated EFL instruction after 2020 has reshaped the conditions for ILP development. However, existing reviews largely predate this pivotal shift and offer only fragmented coverage on how these new ecologies have changed EFL learners’ prospects for ILP development and its assessment. This scoping review provides the first integrative synthesis of research on EFL learners published since 2020. Its primary goal is to consolidate dispersed evidence to identify key developmental patterns and contextual moderators. Four databases (Web of Science, Scopus, ScienceDirect, and ERIC) were searched for publications from 2021 to 2025 using predefined criteria; 56 studies met the eligibility criteria. Reporting follows PRISMA-ScR, and data were independently double-screened, coded, and analyzed thematically in ATLAS.ti 23. The analysis identified seven thematic categories ranging from core learner-internal pragmatic competencies to external pedagogical and computer-mediated contexts. Despite uneven coverage across themes and regions, findings converge on two patterns: pragmalinguistic gains often outpace sociopragmatic appropriateness and follow nonlinear trajectories, and instruction tends to outperform mere exposure when explicit form-function-context mapping, multimodal modelling, and data-driven feedback are provided. For researchers, the review highlights the need to shift toward longitudinal, interaction-sensitive designs that capture within-learner moderators and situational dynamics across broader learner groups. The distinctive contribution is a set of evidence-informed design principles that help educators and materials developers align ILP-oriented instruction and assessment with EFL learners’ varying developmental needs.

## 1. Introduction

In Second Language Acquisition (SLA) research, L2 learners are assumed to build an internalised system that draws on both the target language and the first language, an “interlanguage” [1]. It concerns how learners use language appropriately and effectively across various communicative contexts [2]. Given the nonlinear and variable nature of ILP development, EFL learners struggle with politeness- and facework-sensitive communication [3–5], reflecting their limitations in aligning forms with context-dependent social meanings and relational goals. Digitally mediated environments further complicate these processes [6]. However, while recent empirical studies examine how EFL learners manage social interactions, research has largely overlooked the ILP development under post-pandemic conditions.

Previous reviews clarified developmental mechanisms and estimated instructional effects [7,8], but they largely predate the post-2020 expansion of digitally mediated and multimodal learning ecologies, providing limited coverage across regions and designs. This review, therefore, offers a dedicated scoping synthesis (2021–2025) that maps how ILP development has been evidenced in contemporary EFL contexts. A scoping review is particularly appropriate given the field’s conceptual fragmentation and significant heterogeneity. Following Arksey and O’Malley’s scoping framework [9] and PRISMA-ScR reporting guideline [10], this review charts the range of evidence and highlights gaps rather than conducting meta-analytic pooling. It aims to inform instruction and assessment while orienting future empirical research. This research addresses the following questions:

RQ1: What are the patterns and trends in research on EFL learners’ ILP development, considering journal sources, country of origin, and publication timeline?

RQ2: What are the research priorities of EFL learners’ ILP development, and how are they evidenced in definition/operationalization, proficiency links, instruction, and other intervening factors?

## 2. Literature review

**Fig 1:**
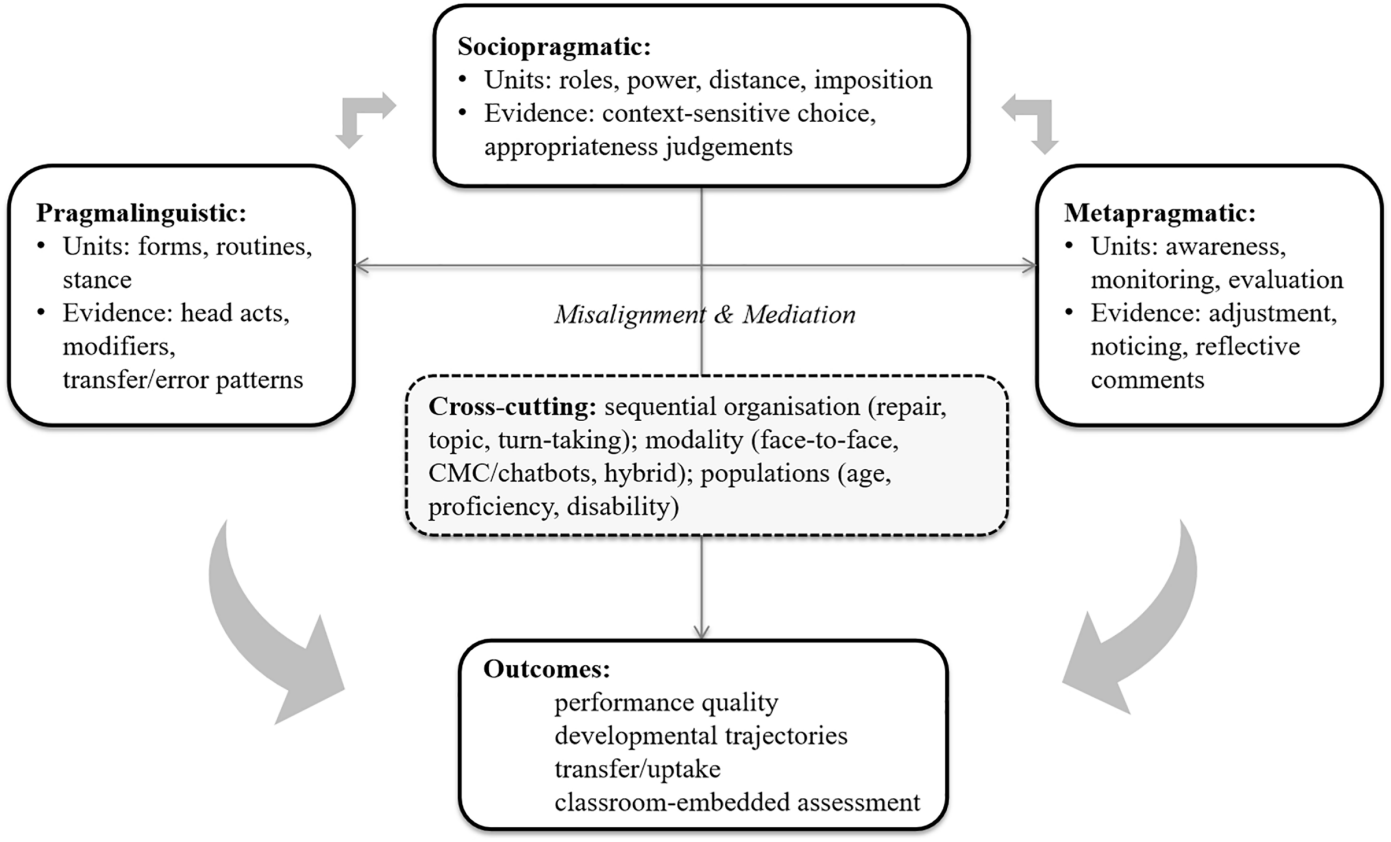
Diagram of ILP concepts and interrelations.

ILP studies are mainly about how L2 learners use, interpret, and evaluate action in interaction across the following competence domains: pragmalinguistic competence (form-function resources) [11–13], sociopragmatic competence (contextual appropriateness and normative appropriateness [14,15], and metapragmatic competence (awareness and evaluation of pragmatic choices) [16,17]. However, these domains are often studied in relative isolation, which can obscure how misalignment arises (e.g., rich forms without sociopragmatic fit) and how cross-cutting factors shape pragmatic opportunities in face-to-face and digitally mediated interactions. Fig 1 summarizes the conceptual links among these domains and provides the basis for the outcome categories charted in this review, including performance quality, developmental trajectories, transfer or uptake, and classroom-embedded assessment.

Across studies, ILP development is examined from micro-interactional organisation to meso-classroom/task configurations and macro-sociocultural expectations. Earlier reviews mapped key issues but relied mainly on classroom- and speech-act-oriented evidence. This scoping review foregrounds meso-level links between observed ILP performance and contextual arrangements, drawing on micro-analytic and macro-level insights to interpret developmental patterns under contemporary, digitally mediated learning conditions. Table 1 summarizes major prior reviews and clarifies the added value of this scoping review.

**Table 1:** Major ILP development reviews and the added value in this scoping review.

Source	Scope & Coverage	Key takeaways	Added value
Kasper & Schmidt (1996) [18]	Profiles ILP as an SLA subfield with a developmental lens; surveys evidence on learning, mechanisms, and theory links	Frames core developmental issues, connects ILP findings to SLA theory; early corpus, heavy speech-act	Anchors historical baselines; enables comparison between foundational agenda and contemporary expansions (sequential/interactional, modality, assessment)
Kasper & Rose (1999) [19]	Field-level review of ILP with emphasis on learning/development and proficiency effects; synthesizes evidence	Highlights developmental trajectories and methodological constraints of the 1990s; calls for diversified designs	Clarifies where evidence has strengthened and where gaps persist
Taguchi (2011) [20]	Instructional approaches to L2 pragmatics; explicit/implicit teaching, tasks, feedback, and outcomes	Summarizes instructional effectiveness and constraints; limited alignment between measures and developmental inference	Aligns instructional design, participation structures, and assessment choices with development-oriented mapping
Roever (2011) [21]	Constructs, task formats, scoring, validity/reliability in L2 pragmatics assessment	Identifies construct under-representation and threats to validity/reliability	Connects assessment choices (e.g., judgment/justification) to observed performance and developmental claims
Bardovi-Harlig (2013) [22]	State-of-the-art review on L2 pragmatic development across settings, features, and evidence types	Contrasts the ordering of socio- vs pragmalinguistic development; emphasizes design sensitivity and diverse evidence	Maps convergence/tension across contexts; foregrounds moderators (proficiency, cognition, affect, setting) to inform synthesis
Taguchi (2015) [23]	Instructed pragmatics; task types, contexts, and assessment tendencies	Heterogeneous tasks/outcomes complicate comparisons; calls for clearer operationalization	Code operationalization and links them to trajectory types and context-sensitive outcomes

### 2.1. Interlanguage pragmatic competence

Building on the interlanguage perspective, this section evaluates ILP competence evidence across use, context, and awareness. Traditionally, ILP competence research prioritized form-function mappings, with speech acts serving as the primary evidential base. However, focusing exclusively on discrete acts can obscure how sequential organization, prosody, and interactional contingencies shape action in situ [13,14,24,25]. Consequently, recent work highlights cross-cultural mediation [26] and the role of metapragmatic monitoring [12,15,27,28]. Since interactional actions are jointly constructed [4], L2 learners require meta-awareness to navigate cultural differences [29]. Crucially, existing competence constructs rely predominantly on data from single-language or Asian EFL classrooms, a narrow validation that makes it challenging to distinguish cross-contextual from context-bound features. These limitations motivate the present review’s focus on how ILP is defined and warranted across diverse populations, settings, and modalities.

### 2.2. Interlanguage pragmatic development

Distinct from competence, ILP development focuses on the process by which L2 learners acquire the capacity to perform communicative actions over time. While longitudinal research has expanded, coverage remains uneven across settings and measures [26]. Typically, L2 learners move from an early reliance on L1 transfer toward a gradual refinement of context-sensitive use [18]. However, because pragmatic features develop at different rates [30], analyzing outcomes alone is insufficient; valid inferences require tracing the developmental process itself, including repair and contingencies. Moreover, individual trajectories are significantly shaped by variations in proficiency, affect, and cognition [31–33]. These factors necessitate pedagogies that align tasks and feedback with specific pragmatic demands. Yet, because much evidence relies on short-term interventions with homogeneous cohorts, claims regarding durability and transferability remain constrained.

Scholars remain divided over the sequencing of ILP development. One line of research suggests that learners consolidate pragmalinguistic routines before mastering sociopragmatic calibration [13,18]. Conversely, research in academic settings indicates that sociopragmatic awareness emerges first (e.g., deference, role relations), while learners struggle to mobilize the corresponding linguistic resources [29,34]. Evidence from study-abroad contexts suggests a more complex reality: while simple routines stabilize quickly, context-sensitive choices, such as stance-taking, evolve through slower, nonlinear trajectories [24,35]. These findings indicate that there is no single universal sequence; instead, development follows multiple, context-sensitive pathways conditioned by task design, register, feedback, and participation structures.

To capture these complex trajectories, the field is moving beyond representational models toward interaction-centred approaches [18,24,25,35]. This shift is particularly evident in digital EFL environments, where archived interactions allow learners to revisit and assess their own pragmatic development. However, despite these methodological advances, the evidence base remains skewed. Critical populations (e.g., young learners, students with disabilities, and lower-proficiency cohorts) and specific instructional settings (e.g., EMI and CLIL) are significantly underrepresented. This limited demographic and contextual coverage constrains the generalizability of current findings. Consequently, this review aims to map these disparities, identifying where cumulative evidence is robust and where targeted inquiry is needed.

## 3. Research methods

**Fig 2:**
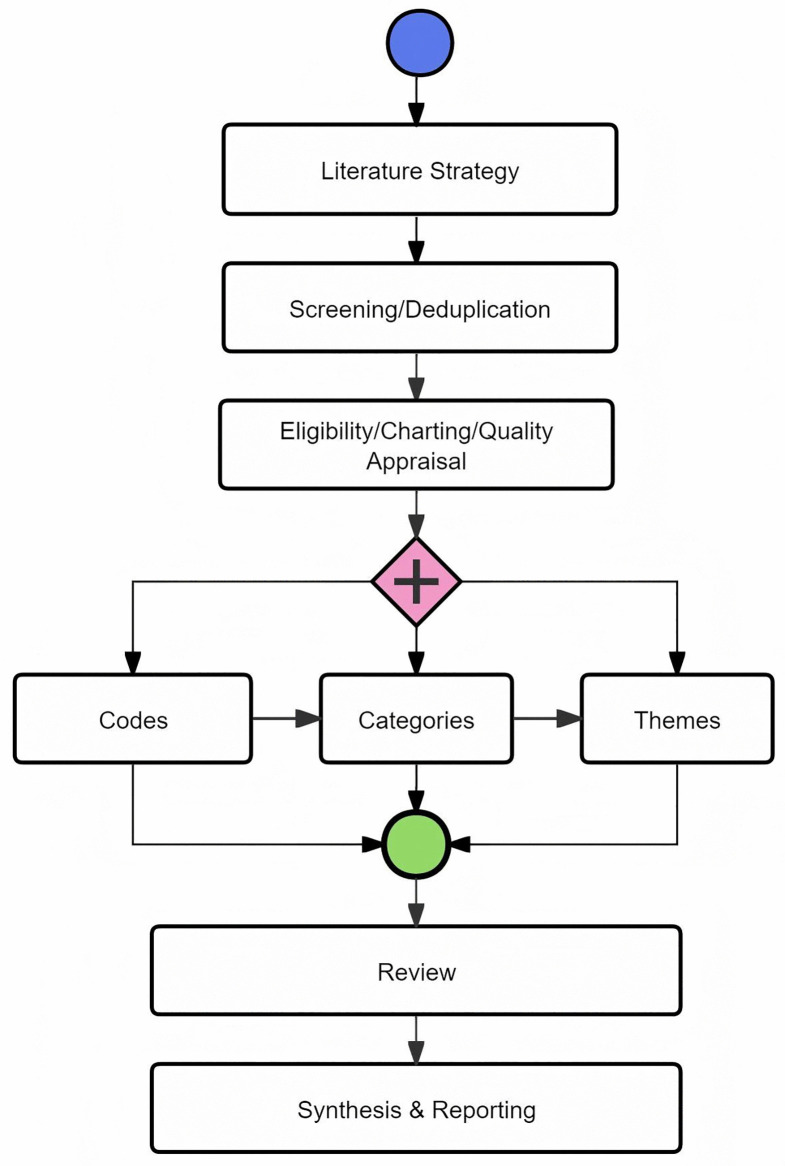
Methodological workflow.

This study employs a scoping review design to synthesize prior research, delineate key thematic domains, and identify gaps. The primary analytical technique is the thematic grouping of findings from eligible studies, a method applied in previous scoping reviews on related topics [28,36]. Identification and screening decisions are reported in accordance with PRISMA-ScR [10], using a flow diagram adapted from the PRISMA 2020 template [37]. To enhance transparency, Fig 2 summarises the methodological workflow from protocol development to synthesis. A review protocol was developed and registered with the International Platform of Registered Systematic Review and Meta-analysis Protocols (INPLASY; DOI: 10.37766/inplasy2024.8.0032). The DOI resolves via the International DOI Foundation’s DOI system to the publicly accessible protocol record (https://www.doi.org).

### 3.1. Literature search

The literature search was conducted between 15 and 17 March 2025 and updated on 8 July 2025, across four international databases: Web of Science, Scopus, ERIC, and ScienceDirect (Table 2). The search was restricted to peer-reviewed journal publications within a specified timeframe. A standardized set of search terms was applied across databases, combining “interlanguage” with learner descriptors (“EFL learners”, “English learners”, “English as a foreign language”). In Web of Science, this query was applied to the Topic Search (TS) field. In Scopus, the same Boolean logic was applied to the TITLE-ABS-KEY field. Equivalent phrases were used for keyword searches in ScienceDirect and ERIC, with manual filters applied where Boolean functionality was restricted. The search strings and the number of records retrieved from each database are summarized in Table 2.

**Table 2:** Search strings from Web of Science, Scopus, ERIC, and ScienceDirect.

Name of Database	Search String (s)	Number
Web of Science	TS=(“interlanguage” AND (“EFL learners” OR “English learners” OR “English as a foreign language”))	58
SCOPUS	TITLE-ABS-KEY (“interlanguage”) AND (TITLE-ABS-KEY (“EFL learners”) OR TITLE-ABS-KEY (“English learners”) OR TITLE-ABS-KEY (“English as a foreign language”))	91
ERIC	“interlanguage” AND (“EFL learners” OR “English learners” OR “English as a foreign language”)	30
ScienceDirect	“interlanguage” AND (“EFL learners” OR “English learners” OR “English as a foreign language”)	152

**Fig 3:**
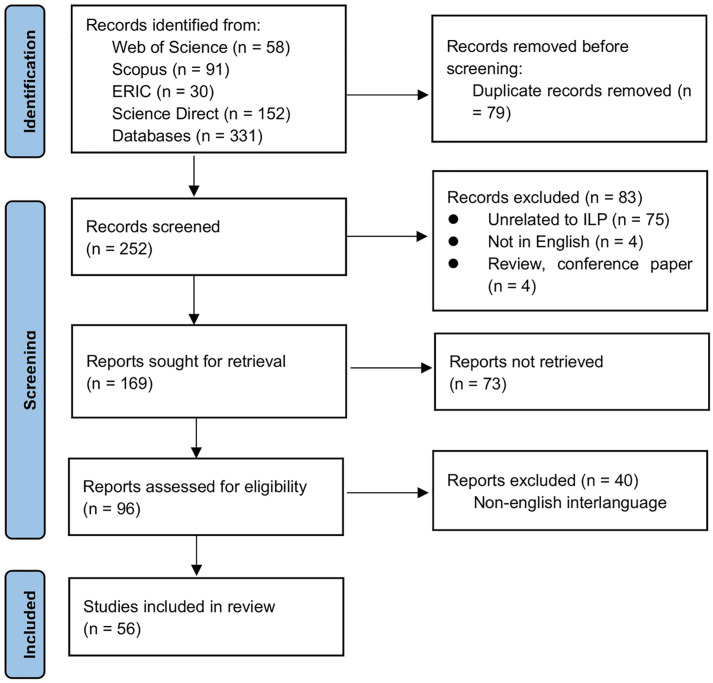
PRISMA-ScR flow diagram of study screening (adapted from the PRISMA 2020).

The initial search yielded 58 records from Web of Science, 91 from Scopus, 30 from ERIC, and 152 from Science Direct. Records were exported to a reference manager, where duplicates were removed. Titles, abstracts, and full texts were then screened against the inclusion and exclusion criteria; studies that were not empirical ILP research, did not focus on EFL/ESL learners, or lacked an accessible full text were excluded. After de-duplication and screening, 56 articles were retained for synthesis (Fig 3). Two reviewers independently screened all titles/abstracts and full texts to ensure consistency during screening and data extraction. Inter-rater reliability was substantial: Cohen’s kappa (κ) = 0.78 (95% CI, 0.72–0.84) for title–abstract screening and κ = 0.82 (95% CI, 0.76–0.88) for full-text decisions; overall percent agreement was 88%. Disagreements were resolved by consensus.

### 3.2. Inclusion and exclusion criteria

To maintain methodological rigor and verifiability, we exclude grey literature (theses, dissertations, conference abstracts, preprints, and industry reports), as these sources often lack standardized peer-review processes. Consistent with established scoping review protocols, non-English records are also excluded to avoid translation-related distortion and ensure coding consistency. However, the epistemological trade-offs of this decision must be explicitly acknowledged. This restriction risks filtering ILP development through Anglophone norms, potentially obscuring indigenous theoretical frameworks. Maximum language representativeness is here subordinated to the necessity for methodological consistency and transparency. Studies were included if they met the following criteria: (1) contained keywords such as “English as a Foreign Language (EFL)” or “English as a Second Language (ESL)” and “interlanguage pragmatics” or “interlanguage pragmatic development”; (2) addressed at least one of the review questions; and (3) focused on English language learning. These criteria serve to ensure conceptual consistency in examining the ILP development of EFL learners.

### 3.3. Study quality assessment

A standardized data charting form was developed in Microsoft Excel to extract relevant information from each study. Prior to formal charting, calibration rounds were conducted on typical and borderline cases to refine a shared coding protocol. The evidence base was descriptively assessed using Crowe’s Critical Appraisal Tool (CCAT), selected for its versatility in accommodating diverse study designs, including mixed-methods, qualitative, and quantitative research. Scoring adhered to the detailed criteria outlined in the *CCAT User Guide* (S1 Table). In this scoping review, CCAT ratings were used descriptively to verify minimum reporting quality and to inform the narrative synthesis. Total CCAT scores were calculated for each study, with the majority ranging from 35 to 40 (M ≈ 36.6). Discrepancies were resolved through consensus discussions among the first author and co-authors. The final charted dataset, covering these core fields for each included study, is presented in S2 Table.

### 3.4. Analysis and synthesis

Bibliographic management and classification were conducted in ATLAS.ti 23. Following Zairul (2020) [38], articles were classified by author, journal, issue, publisher, volume, and year to facilitate temporal and thematic exploration of the dataset. In the qualitative phase, thematic analysis was performed in accordance with Braun and Clarke (2006) [39] and Alyaqoub et al. (2024) [40], involving familiarisation, coding, theme generation, review, definition, and reporting. Consistent with the mapping aim of a scoping review, no meta-analysis was undertaken. Evidence was synthesized narratively through thematic analysis and data charting.

The coding process adopted the hybrid inductive-deductive approach described by Aslam et al. (2023) [41] and Rasool et al. (2023) [42], balancing theoretical coherence with data-driven sensitivity. Prior to the main coding phase, pilot coding was conducted to align interpretive perspectives. Subsequently, two coders independently completed the initial coding, achieving a substantial inter-coder reliability (κ > 0.73). Disagreements were resolved through iterative discussions to ensure interpretive alignment, with a third reviewer adjudicating intractable items. Reflexive memos were maintained in ATLAS.ti to document boundary decisions for sentence- or clause-level meaning units, thereby establishing a transparent and auditable decision trail.

Initial line-by-line coding was conducted using the six levels of pragmatic analysis proposed by Barron (2019) [24]: formal, actional, interactional, topic, organizational, and stylistic. Within this deductive framework, inductive coding was carried out to capture emergent sub-themes. Through this recursive process, codes were rigorously reviewed; for instance, initial groupings of speech acts and linguistic strategies, at Barron’s formal and actional levels, were subsequently divided into distinct themes to highlight their differing analytical emphases. The final themes include: speech acts, linguistic strategies and features, pragmalinguistic competence and development, sociopragmatic competence and intercultural communication, metapragmatic competence, pedagogical context, and computer-mediated communication (CMC).

## 4. Results and discussion

The findings are presented through a combination of descriptive statistics and qualitative thematic analysis. While RQ 1 is addressed in the quantitative mapping of the dataset, RQ 2 is explored in the qualitative synthesis. With a minimum token frequency threshold of 343 in ATLAS. ti 23, the top ten keywords’ token frequencies reveal the most prominent concepts related to EFL learners’ ILP development (Table 3). These frequencies represent the lexical salience of what the literature most frequently “talks about” rather than the conceptual depth or theoretical sophistication of work on each construct. The subsequent thematic analysis in Section 4.2 serves to complement these findings by examining how the literature substantively do with those constructs.

**Table 3:** Token frequency of the top ten words.

Rankings	Keywords	Token Frequency
1	language	3957
2	learners	3280
3	L2	2489
4	study	1916
5	pragmatic	1750
6	use	1507
7	strategies	1412
8	pragmatics	1382
9	speech	1220
10	participants	1026

**Fig 4:**
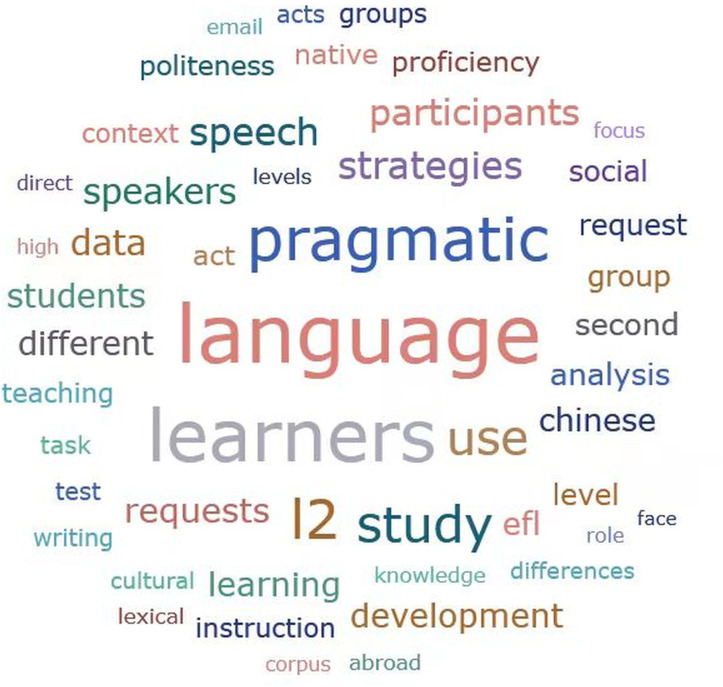
Word cloud and word frequencies. Font size corresponds to word frequency.

The keyword profile underscores a learner-centered and usage-oriented empirical foundation, with a recurrent emphasis on ILP development as manifested in speech-act performance and strategic language use. The prominence of terms such as “study” and “participants” suggests that a substantial portion of the corpus is operationalized through design-driven empirical methodologies. This finding aligns with the observation that interventionist and classroom-based research continue to exert significant methodological influence within ILP inquiry. These patterns highlight a sustained focus on the mechanisms through which learners deploy pragmatic resources in context and their developmental trajectories over time. Below is the word cloud for the terms related to EFL learners’ ILP development across all reviewed articles (Fig 4).

### 4.1. Statistical descriptions

This subsection directly addresses RQ1 by describing outlet patterns, country-of-origin distributions, and thematic trends between 2021 and 2025. The reviewed studies are distributed across a range of journals, as detailed in Table 4. *Journal of Pragmatics* and *System* account for nearly one-third of the corpus (17.9% and 16.1%, respectively), signifying a pronounced concentration of ILP development work in these two applied linguistics journals. Beyond this concentration, the long tail of outlets suggests that empirical evidence is institutionally dispersed rather than confined to a small set of specialist periodicals. Among them, journals such as *English for Specific Purposes*, *Computers & Education*, and *Computer Speech and Language* reflect the multidisciplinary breadth of ILP research. This dispersion suggests that knowledge production in the field is shaped by multiple editorial traditions, a factor that may partially explain the observed heterogeneity in constructs, methodological measures, and reporting practices across the sampled studies.

**Table 4:** Journals and publication years.

	2021	2022	2023	2024	2025	Totals
Journal of Pragmatics	3	2	5	0	0	10
System	3	1	1	2	2	9
Assessing Writing	1	2	0	0	0	3
English Language Teaching	1	1	0	0	0	2
Research Methods in Applied Linguistics	0	0	0	1	1	2
Lingua	2	0	0	0	0	2
Learning and Motivation	0	0	0	2	0	2
Language Sciences	0	1	1	0	0	2
Language Teaching Research	0	0	0	0	1	1
Pragmatics	1	0	0	0	0	1
Ampersand	0	0	1	0	0	1
The Language Learning	0	1	0	0	0	1
The Electronic Journal for English as a Second Language	1	0	0	0	0	1
Teaching English Language	1	0	0	0	0	1
Linguistics and Education	0	1	0	0	0	1
Journal of Second Language Writing	0	0	1	0	0	1
Journal of English for Academic Purposes	0	0	0	1	0	1
English for Specific Purposes	0	0	0	1	0	1
International Journal of Society, Culture, & Language	0	0	0	1	0	1
Indonesian Journal of Applied Linguistics	1	0	0	0	0	1
Heliyon	0	0	0	1	0	1
Contrastive Pragmatics	0	0	1	0	0	1
Computers & Education	0	0	0	1	0	1
Computer Speech and Language	0	0	0	0	1	1
Journal of Education and Learning	0	0	1	1	0	2
World Journal of English Language	0	0	1	0	0	1
Language Teaching Research Quarterly	0	0	0	1	0	1
Chemistry Education Research and Practice	0	0	1	0	0	1
Language Learning Journal	0	1	0	0	0	1
The Reading Matrix: An International Online Journal	0	0	1	0	0	1
Journal of Language and Linguistic Studies	1	0	0	0	0	1
Total	15	10	15	11	5	56

Further analysis reveals discernible alignment between publication outlets and their respective research orientations. Articles in the *Journal of Pragmatics* predominantly investigate pragmalinguistic [12,28,35] and sociopragmatic aspects of L2 use [43,44], typically grounded in qualitative or discourse-analytic frameworks. Prominent thematic clusters include the realization of speech acts, metapragmatic awareness [17,45], and the negotiation of interactional strategies [46]. Although less pervasive, comparative investigations into instructional paradigms are also represented [47]. In contrast, research disseminated through *System* reflects a more pedagogically anchored and empirically substantiated agenda, frequently leveraging experimental or mixed-methods designs to evaluate the efficacy of instructional interventions [48–50]. Themes such as peer collaboration, computer-mediated learning, and the role of affect are extensively explored, with a sustained focus on classroom-based contexts [51–53]. Given the fragmented distribution of ILP-related studies in other periodicals, definitive trends concerning thematic focus or methodological predilections remain tenuous.

**Fig 5:**
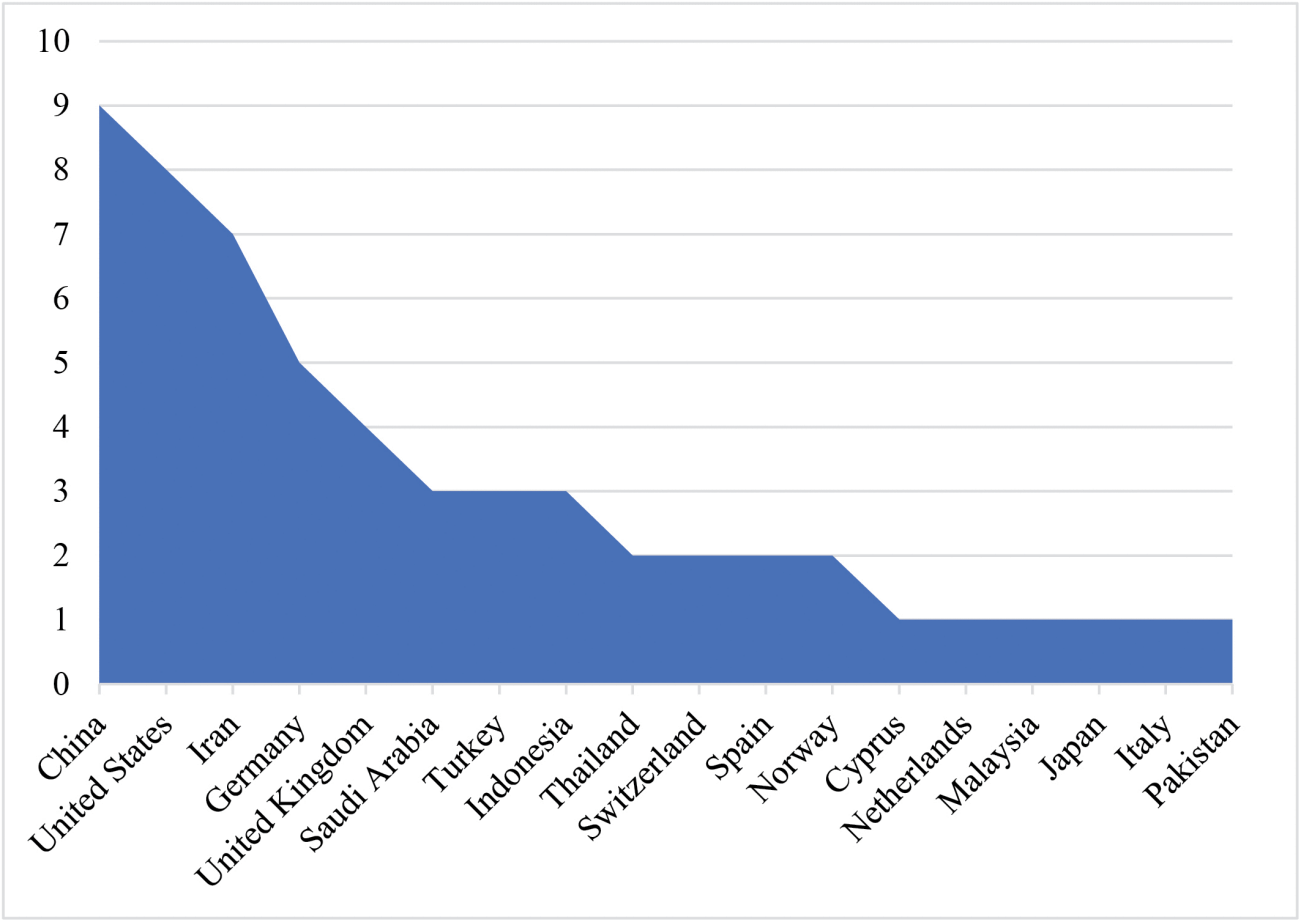
Global distribution of article counts.

The global distribution of article counts reflects widespread scholarly commitment to augmenting EFL learners’ ILP development across heterogeneous linguistic and cultural contexts, as illustrated in Fig 5. While geographically extensive, the distribution remains conspicuously uneven, characterized by a tripartite concentration of high-output regions (notably China, the United States, and Iran) juxtaposed against a multitude of low-output contexts (e.g., Cyprus, Italy, Japan, the Netherlands, Pakistan, and Thailand). This spatial asymmetry carries profound implications: the synthesis is predisposed to be shaped by the dominant instructional settings and learner demographics of high-output contexts, thereby limiting the transferability of empirical claims to underrepresented regions. This imbalance constrains cross-context comparison, insofar as the evidence base in low-output settings is insufficiently granular to determine whether observed patterns are contextually contingent or broadly generalizable. In addition, the observed skew may partially stem from differential visibility of studies within the search strategy (e.g., database indexing and publication language) rather than the definitive absence of research activity.

**Fig 6:**
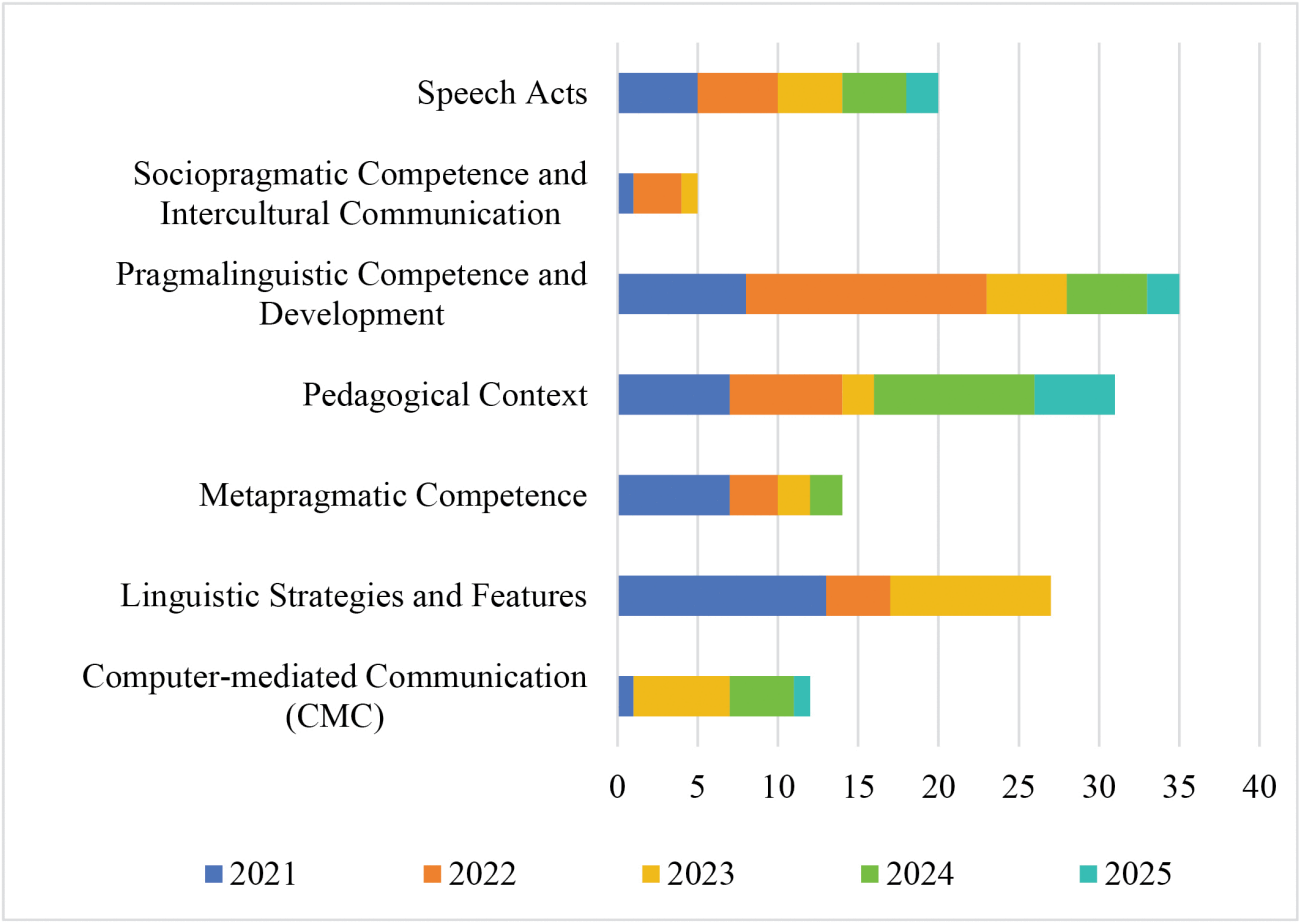
Distribution of ILP themes across publication years (2021–2025). Bars represent the frequency of coded theme occurrences across all 56 studies.

The examination of the seven identified themes reveals distinctive thematic trends in scholarly attention spanning the 2021–2025 period (Fig 6). Given that individual publications frequently intersect multiple themes, the reported frequencies ought not to be treated as direct proxies for the conceptual depth or theoretical sophistication underlying each theme. Instead, these metrics signal specific nodes where empirical inquiry has clustered. Among them, pragmalinguistic competence and development emerged as the most prominent focal point (32 occurrences). In contrast, sociopragmatic competence and intercultural communication remained comparatively peripheral (5 occurrences). The remaining themes occupy the intermediate space between these two poles. This distributional pattern reveals a prevailing research orientation that prioritizes linguistically tractable phenomena and pedagogy-facing inquiries over the complexities of socially situated appropriateness and intercultural calibration.

### 4.2. Qualitative results and discussions

**Fig 7:**
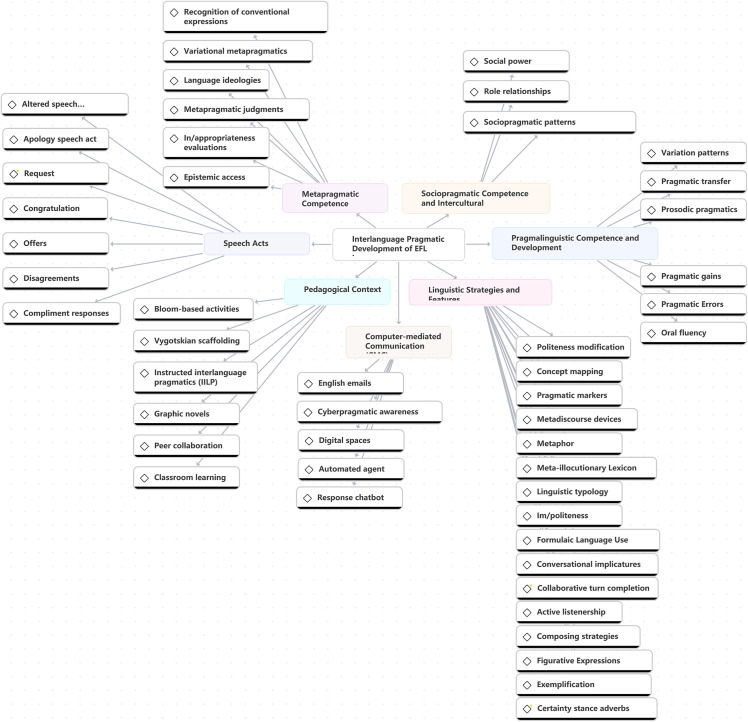
Thematic network of EFL learners’ ILP competence and development.

Since the publication of Selinker’s (1972) *Interlanguage* paper [54], ILP research has evolved toward more explicit measurement and a stronger focus on EFL learner evidence. To address RQ2 explicitly, this section analyzes the reviewed articles through a thematic network (Fig 7). Each theme is discussed in terms of (i) definition (what aspect of ILP develops), (ii) operationalization (how it is typically measured or elicited), and (iii) moderators (factors such as proficiency, task design, and setting). Across themes, points of convergence and divergence are presented as a cumulative interpretation of EFL learners’ ILP development.

The thematic network categorizes findings into four competence domains and two ecological drivers. Speech acts focus on specific realizations, such as apologies and refusals [55]; linguistic strategies and forms serve as distinct resources for adapting these realizations [22]; pragmalinguistic and sociopragmatic competences represent the coordination of linguistic resources with social appropriateness [25]; and metapragmatic competence regulates awareness and strategic control [16,17]. Finally, pedagogical context and computer-mediated communication (CMC) are analyzed as ecological conditions that drive development. Table 5 summarizes how these themes relate to L2 learner challenges, interventions, and outcomes.

**Table 5:** Summary matrix linking themes to L2 learner challenges, instructional interventions, and observed outcomes.

Themes	L2 Learner Challenges	Instructional Interventions	Observed Outcomes
Speech Acts	Pragmatic transfer, L1 influence, and inappropriate realisation [43,56]	Role plays, Bloom-based tasks, and Vygotskian scaffolding [57,58]	Increased pragmatic accuracy and variation [55,59]
Linguistic Strategies	Limited use of pragmatic markers, lexical bundle overuse, and difficulty with figurative language [45,60,61]	Explicit instruction and collaborative interaction [47,62]	Improved fluency, politeness, and alignment [36,63]
Pragmalinguistic & Sociopragmatic Competence	Imbalanced development, overgeneralization, and conceptual transfer [13,27,34]	Usage-based modeling and sociopragmatic sensitivity training [12,64]	Greater coordination of linguistic form and social meaning [15,28]
Metapragmatic Competence	Low awareness of pragmatic appropriateness and ambiguous conceptual scope [27,65]	Metacognitive strategy instruction and genre awareness training [62,66]	Enhanced self-monitoring and reflective judgment [16,17]
Pedagogical Context	Lack of input in textbooks, underrepresentation in curricula, and emotional and cognitive vulnerabilities [50,67]	Task-based models, concept-based instruction, and pair collaboration [35,48,68]	Improved speech act realization, affective and contextual awareness [69,70]
Computer-Mediated Communication	Limited interaction and low engagement in digital environments [71]	Data-driven CMC and AR-enhanced robot learning [49,72]	Improved pragmatic performance and learner participation [71,73]

The analysis below synthesizes these themes, referencing the summary matrix both within themes and across dimensions. Given their reciprocal influence and frequent co-occurrence, pragmalinguistic and sociopragmatic competence are discussed jointly in this section [15,28,34,74]. To address RQ2, the discussion progresses logically from observable pragmatic performance (speech acts, linguistic strategies) to underlying competence constructs that regulate appropriateness (pragmalinguistic, sociopragmatic, and metapragmatic competence), and finally to ecological conditions that shape these developments (pedagogical context, CMC). This sequencing clarifies not only what develops but also how development is evidenced and under what conditions it occurs.

#### 4.2.1. EFL learners’ speech acts.

Most of the included studies define ILP development as the growing ability to perform pragmatic actions in the face of contextual constraints. While learner performance is influenced by variables like social status, distance, and imposition [58], it is heavily conditioned by L1 cultural norms [15,75,76]. For instance, Chinese EFL learners often transfer L1 disagreement patterns into English [59], and high-proficiency Saudi learners frequently adhere to L1 conventions [43]. Yet, despite this persistent L1 pull, evidence suggests that structured activities (e.g., concept mapping, Vygotskian scaffolding) can shift performance toward target-like realization [28,57]. Thus, speech act development involves a tension between resistant L1 baselines and pedagogy-enabled convergence.

Methodologically, this body of research relies heavily on Discourse Completion Tests (DCTs), grounded in Searle’s speech act theory (1969) [77] and Brown and Levinson’s politeness theory (1987) [78]. However, a recurrent critique is that DCTs fail to capture the sequential and interactional nature of communication. In response, recent scholarship advocates supplementing DCTs with role plays and elicited conversations to better approximate real-time processing [79]. This methodological shift highlights a critical gap: understanding how linguistic strategies and features function within dynamic interaction. This interactional dimension moves beyond isolated speech acts, a focus addressed in the next section.

#### 4.2.2. EFL learners’ linguistic strategies and features.

Beyond discrete speech acts, interactants use a repertoire of linguistic strategies to actively demonstrate listenership, thereby asserting and defending epistemic access [62]. This theme covers interactional phenomena, including alignment, pragmatic markers (PMs), and metadiscourse. Research reveals nonlinear developmental trajectories in these areas. Xiao et al. (2021) [60] report non-linear trajectories in PM development, while Savić et al. (2021) [12] note that certain syntactic downgraders remain stable despite proficiency gains, even as age and proficiency increase. Additionally, the uptake of these markers is often modulated by learning conditions, as explicit instruction generally favors acquisition [47]. In sensitive contexts, learners may manage stance through figurative language [45] or by reducing mitigation [63], though evidence regarding the trade-off between politeness and stance clarity remains limited across L1 backgrounds.

Formulaic expressions, particularly lexical bundles, provide complementary evidence of development. Pan (2023) [61] found that higher-proficiency EFL learners produce a smaller and more flexible range of bundles compared to the rigid overuse seen in lower-proficiency peers. Conversely, explicit instruction improves fluency in formulaic sequences across all proficiency levels [74]. In sum, pragmatic competence requires coordinating strategies with formulaic resources. ILP development is marked by a shift from unstable patterns toward flexible repertoires. These resource-level patterns underscore the explanatory need for pragmalinguistic and sociopragmatic competence, which is addressed in the next section.

#### 4.2.3. Pragmalinguistic and sociopragmatic competence and development.

This theme examines ILP development as the coordinated evolution of form–function mappings (pragmalinguistic) and sociocontextual appropriateness (sociopragmatic). Since Leech (1983) established this distinction [80], scholars have debated their developmental trajectory. While early accounts questioned whether pragmalinguistic resources precede sociopragmatic sensitivity [13] or the reverse [34]. Recent longitudinal evidence points to asynchronous co-development [81]. For instance, Hao et al. (2021) [82] used dependency-direction metrics to track typological features and showed that some form choices converge, while others plateau. Furthermore, persistent constraints such as the erroneous use of high-frequency verbs reveal systematic interlanguage differences [83,84]. These studies indicate that form-level preferences may resist change even as general proficiency increases, creating a risk of misalignment with sociopragmatic expectations.

Turning to sociopragmatic determinants, recent scholarship examines how L2 learners operationalize social variables across proficiency levels [11,74]. Eslami et al. (2022) [15] report status-driven patterns in perceptions of offence, obligation to apologise, face loss, and apology acceptance across proficiency bands, while Alshraah (2024) [64] observes that higher-proficiency learners display greater sensitivity to power dynamics in requests. From a cross-linguistic perspective, Napoli and Tantucci (2022) [28] demonstrate that social distance effectively predicts the use of indirectness strategies. Despite these gains, contextual sensitivity can remain limited in ILP development. Current scholarship thus positions metapragmatic competence as the essential regulatory mechanism for bridging this gap. Recognizing its role in monitoring and refining performance, the following section explores how this metapragmatic awareness develops and functions.

#### 4.2.4. Metapragmatic competence.

Recent work foregrounds metapragmatic competence as a key driver of ILP development, distinguishing it from mere performance [79]. While traditional definitions outline metacognitive, metarepresentational, and metacommunicative dimensions [16], Hübler (2011) [65] argues that the term often suffers from conceptual ambiguity. To resolve this, this review adopts a working definition: metapragmatic competence is the awareness, evaluation, and strategic control of pragmatic action. It functions as the cognitive interface that connects a strategy to its linguistic realization [16,65]. By linking social judgment with linguistic choice, this competence allows learners to monitor their output and stabilize the fluctuations often observed in interlanguage development. This self-regulatory capacity is driven by learner autonomy, which has been shown to increase the deployment of Interlanguage Pragmatic Learning Strategies (IPLS) [85].

Empirically, this competence manifests in how learners label and judge communicative acts, though patterns vary by register. Schneider (2021, 2022) documents that meta-illocutionary expressions differ significantly across varieties [17,86]. At the learner level, Saleem and Saleem (2023) [27] analyzed student-faculty emails and found that advanced learners primarily rely on criteria of formality and structural framing to justify their appropriateness judgments, often overlooking relational variables. This heavy reliance on formality underscores the complexity of developing intergeneric awareness in digital contexts [87]. However, research frequently conflates metapragmatic knowledge with metalinguistic knowledge and relies on self-reports without coded justifications [88]. Given that this competence is highly responsive to environmental input, a significant body of research investigates pedagogical contexts that shape its development, as detailed in the following section.

#### 4.2.5. Pedagogical context.

Fostering EFL learner autonomy requires specifying effective instructional strategies [89]. Instructed Interlanguage Pragmatics (IILP) investigates how teaching interventions facilitate the mapping of linguistic forms to social functions [3,71]. Theoretically, IILP draws on the noticing and interaction hypotheses, which model how learners consolidate pragmatic features through noticing, practice, and cognitive processing [90]. While early research focused on the superiority of explicit over implicit instruction [91], recent scholarship suggests this binary overlooks contextual nuances. Critics argue that a sole focus on explicit rules may ignore the negotiation of meaning and linguistic variability required in real-world interaction [35]. Consequently, current research has shifted from asking whether instruction works to defining the specific conditions under which it is most effective.

Empirical evidence indicates that instructional effectiveness is driven by task design, proficiency alignment, and modality. Explicit and task-based designs consistently yield gains [69], though effects vary by proficiency: lower-proficiency learners benefit from Bloom-Vygotskian scaffolded, routine-based practice [57,68], whereas advanced learners improve through multi-turn tasks requiring reflection [35]. Beyond individual tasks, interactional formats such as pair work facilitate the acquisition of implicature [48]. Moreover, multimodal inputs broaden opportunities, as graphic novels have been used pedagogically [92], while function-specific work extends coverage [93].

However, uniform interventions often yield divergent outcomes due to individual and relational moderators. In the affective domain, learner vulnerability constrains the uptake unless teachers employ specific regulatory strategies [50]. Relationally, the quality of teacher-student rapport amplifies or dampens instructional gains, with teacher preparedness playing a critical role in the quality of feedback [52,70]. On the cognitive side, factors such as attentional control and cognitive load mediate the processing of explicit input [53,94]. This indicates that while aligning form, function, and context is foundational, instructional efficacy depends on the learner’s affective, relational, and cognitive resources.

Despite these findings, a significant implementation gap persists**.** Instructional materials frequently reduce pragmatics to static formulas, obscuring the dynamic negotiation of meaning and sociopragmatic variation [35]. Corroborating this systemic deficit, Siddiqa and Whyte (2025) [67] reveal that pragmatic input is not only scarce but functionally limited, with even advanced-level texts failing to model complex contextual parameters. To bridge this divide, the field must pivot from static method comparisons to generative, dynamic instructional design. As digital environments constitute a primary domain for such inquiry, the following section investigates the ecological role of Computer-Mediated Communication (CMC) in ILP development.

#### 4.2.6. Computer-mediated communication.

Building on the pedagogical shift toward digital environments, Computer-mediated Communication (CMC) represents a distinct ecological condition for ILP development. Digital platforms expand communicative affordances, offering new participation structures beyond the classroom [95,96]. It has been proven that developmental outcomes hinge on mode and scaffolding: synchronous and asynchronous CMC yield different pragmatic gains [95], while well-structured tasks within online communities enhance acquisition [44,63]. However, efficacy is maximized when CMC is coupled with explicit instruction rather than used in isolation. For instance, integrating CMC with data-driven feedback outperforms unguided interaction [49]. Rafiq and Yavuz (2024) [71] confirmed that both implicit and explicit online training improved learners’ pragmatic performance, with comparable effects across delivery modes. Beyond standard CMC, emerging technologies expand the toolkit: AI-based surprisal measure now assesses lexical complexity [73], while augmented reality in robot-assisted learning targets domain-specific pragmatics [72].

Yet, these benefits are not intrinsic to the medium. There is no uniform “more CMC equals better ILP” effect; instead, outcomes are contingent on task design and interactional mode. CMC functions less as an inherently effective method than as a context where design features moderate ILP development. The research focus has thus shifted from whether CMC works to how specific configurations of tools, tasks, and feedback facilitate acquisition. Research has demonstrated that virtual exchange alone fails to guarantee exposure to native-like patterns [97]. Similarly, interactions with automated agents shape pragmatic behavior differently than human interlocutors, revealing inherent design constraints [46,98].

Section 4.2 synthesizes the evidence to characterize ILP development not as a linear accumulation of forms, but as a dynamic negotiation constrained by how constructs are defined and operationalized. The persistent imbalance between pragmalinguistic forms and sociopragmatic appropriateness reflects constraints in learning environments that privilege stable input over interactional variability. Accordingly, the field is advancing beyond binary method comparisons toward a granular account of ecological validity, recognizing that learner profiles and degrees of autonomy modulate instructional efficacy. Future inquiry should therefore adopt mechanism-focused designs in which developmental claims are substantiated by explicitly specified constructs, measurement practices, and boundary conditions.

### 4.3. Research gaps

Despite the expansion of ILP research in EFL settings, three primary gaps continue to constrain the evidential basis for pragmatic development. First, the field lacks robust longitudinal evidence. Within-learner change is rarely traced through repeated-measures designs or advanced developmental modelling [99], leaving a reliance on cross-sectional snapshots that cannot fully capture the nonlinear nature of pragmatic growth. Second, sociopragmatic competence remains under-operationalized, particularly regarding evidence-indexical measurement. Few studies use dual-response tasks that pair appropriateness judgments with qualitative rationales and link these to production data, while reporting coding and reliability indices [11,88]. Moreover, the methodological comparability of DCTs, role-plays, and naturalistic interactional data remains under-examined [100]. This lack of comparative rigor complicates the synthesis of cross-study evidence and leaves the field without a unified standard for evaluating pragmatic evidence.

Third, the empirical base is characterized by modality and explanatory fragmentation. Research remains skewed toward spoken interaction, while multimodal sequential organization and computer-mediated communication (CMC) are comparatively under-described [95,96]. Explanatory coverage is further limited by restricted sampling frames, as cognitive, affective, and relational factors are seldom treated as internal moderators of instructional outcomes [50,53,70,94]. This lack of representative diversity is particularly evident among underrepresented populations in English-Medium Instruction (EMI) and Content and Language Integrated Learning (CLIL) settings, which undermines the generalizability of empirical claims across different educational ecologies. Addressing these imbalances requires a transition toward triangulated, multimodal designs that can more holistically capture ILP development across diverse settings.

These persistent gaps necessitate a staged research agenda that moves beyond descriptive accounts toward integrated, multi-dimensional inquiry. Longitudinal mixed-methods designs are required to track intra-individual trajectories while triangulating judgment and production data from the perspectives of L2 learners. Furthermore, measurement research must advance through explicitly articulated validity arguments that link constructs to task design and support cross-task comparability. To extend coverage and scope, multimodal conversation analysis of classrooms, study-abroad programs, and EMI/CLIL is essential to document how speech, prosody, gaze, and other embodied resources jointly realise pragmatic actions. Together, these approaches will operationalize ILP development more holistically, strengthen the evidential basis for measurement choices, and refine the scope of claims across diverse settings.

## 5. Conclusion

This scoping review offers a distinctive contribution to ILP development by providing a systematic synthesis of post-2020 evidence and clarifying the intricate links among construct definition, operationalization, and developmental warrants. The evidence base is thematically uneven and geographically concentrated. The originality of this work lies in its rigorous, PRISMA-aligned mapping of a geographically and institutionally dispersed corpus, revealing that the reported development is consistently context-sensitive, with pragmalinguistic gains more robust than the stabilization of sociopragmatic appropriateness. This synthesis highlights a prevailing research orientation that prioritizes linguistically tractable phenomena over socially situated complexity and intra-individual moderators. Methodologically, the review demonstrates a registered and software-supported workflow that can be adapted in future work.

For pedagogical practice, the findings advocate for proficiency-sensitive design principles rather than a uniform instructional approach. Proficiency conditions implementation: while early-stage learners benefit from scaffolded, routine-supported practice, advanced learners require genre- and interaction-sensitive tasks that elicit rationales and support strategy control. Across levels, effective designs make form-function-context mappings explicit and align tasks and feedback with learner needs. However, the transferability of these implications is currently limited by the regional concentration of studies and the exclusion of non-English publications, which carries significant epistemological implications for the universality of the findings.

Future research needs to orient toward stronger developmental evidence and more comparable measurement. This agenda involves longitudinal within-learner designs to track the nonlinear trajectories of ILP development. In particular, sociopragmatic competence is effectively operationalized through dual-response judgment tasks that pair appropriateness ratings with qualitative rationales, thereby strengthening the validity arguments for measurement choices. Beyond controlled tasks, empirical claims gain persuasiveness when triangulated with naturalistic interaction and analyzed via multimodality-sensitive methods. Finally, moderator modelling of cognitive, affective, and relational factors remains essential for the conditions under which instructional designs remain effective.

While this review offers systematic insights into post-2020 ILP research, certain limitations should be acknowledged. This synthesis is limited by English-only coverage, uneven database access, and geographic concentration (notably East Asia and the Middle East). In addition, learner populations were unevenly represented, as the scarcity of research on young learners and those with disabilities limits the scope of claims regarding age-related trajectories and educational accessibility. Future reviews should establish multi-database search protocols that encompass diverse languages and prioritize underrepresented samples to enhance empirical generalizability. ILP development should be conceptualized as a nonlinear, within-learner process conditioned by myriad internal and external moderators. Within this framework, sociopragmatic appropriateness warrants increasingly rigorous scrutiny to ensure that the field moves toward a more inclusive and ecologically valid understanding of ILP development.

## Supporting information

S1 Table Crowe Critical Appraisal Tool. (DOCX)

S2 Table Data Extraction and Charting of 56 Articles. (DOCX)
