# Supplementary material for: A scoping review of EFL learners’ interlanguage pragmatic development
Source: PLoS One. 2026 Mar 13;21(3):e0344811. doi: 10.1371/journal.pone.0344811 (PMC12987429; doi:10.1371/journal.pone.0344811)
Supplement: S1 Table — (DOCX) [file pone.0344811.s001.docx]

S1 Table. Crowe Critical Appraisal Tool

| Category  Item | Item descriptors  [ 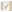Present; 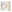Absent; ■ Not applicable] | | | | Description  [Important information for each item] | | | | Score  [0–5] | |  |
| --- | --- | --- | --- | --- | --- | --- | --- | --- | --- | --- | --- |
| 1. Preliminaries | | | | | | | | | | |  |
| Title | | | 1. Includes study aims ❏ and design ❏ | | | |  | | | |  |
| Abstract | | | 1. Key information ❏ | | | |  | | | |  |
| (assess last) | | | 2. Balanced ❏ and informative ❏ | | | |  | | | |  |
| Text  (assess last) | | | 1. Sufficient detail others could reproduce ❏  2. Clear/concise writing ❏, table(s) ❏, diagram(s) ❏, figure(s) ❏ | | | |  | | | |  |
|  |  | | | | **Preliminaries [/5]** | | | |  | |  |
| 2. Introduction | | | | | | | | | | |  |
| Background | | | 1. Summary of current knowledge ❏  2. Specific problem(s) addressed ❏ and reason(s) for addressing ❏ | | | |  | | | |  |
| Objective | | | 1. Primary objective(s), hypothesis(es), or aim(s) ❏  2. Secondary question(s) ❏ | | | |  | | | |  |
|  | **Is it worth continuing?** | | | | **Introduction [/5]** | | | |  | |  |
| 3. Design | | | | | | | | | | |  |
| Research design | | | 1. Research design(s) chosen ❏ and why ❏  2. Suitability of research design(s) ❏ | | | |  | | | |  |
| Intervention,  Treatment, Exposure | | | 1. Intervention(s)/treatment(s)/exposure(s) chosen ❏ and why ❏  2. Precise details of the intervention(s)/treatment(s)/exposure(s) ❏ for each group ❏ 3. Intervention(s)/treatment(s)/exposure(s) valid ❏ and reliable ❏ | | | |  | | | |  |
| Outcome, Output, | | | 1. Outcome(s)/output(s)/predictor(s)/measure(s) chosen ❏ and why ❏ | | | |  | | | |  |
| Predictor, Measure | | | 2. Clearly define outcome(s)/output(s)/predictor(s)/measure(s) ❏  3. Outcome(s)/output(s)/predictor(s)/measure(s) valid ❏ and reliable ❏ | | | |  | | | |  |
| Bias, etc | | | 1. Potential bias ❏, confounding variables ❏, effect modifiers ❏, interactions ❏ 2. Sequence generation ❏, group allocation ❏, group balance ❏, and by whom ❏ 3. Equivalent treatment of participants/cases/groups ❏ | | | |  | | | |  |
|  | **Is it worth continuing?** | | | | **Design [/5]** | | | |  | |  |
| 4. Sampling | | | | | | | | | | |  |
| Sampling method | | | 1. Sampling method(s) chosen ❏ and why ❏  2. Suitability of sampling method ❏ | | | |  | | | |  |
| Sample size | | | 1. Sample size ❏, how chosen ❏, and why ❏  2. Suitability of sample size ❏ | | | |  | | | |  |
| Sampling protocol | | | | 1. Target/actual/sample population(s): description ❏ and suitability ❏  2. Participants/cases/groups: inclusion ❏ and exclusion ❏ criteria  3. Recruitment of participants/cases/groups ❏ | | | |  | | | |
|  | | **Is it worth continuing?** | | | | **Sampling [/5]** | | | |  | |
| 5. Data collection | | | | | | | | | | | |
| Collection method | | | | 1. Collection method(s) chosen ❏ and why ❏  2. Suitability of collection method(s) ❏ | | | |  | | | |
| Collection protocol | | | | 1. Include date(s) ❏, location(s) ❏, setting(s) ❏, personnel ❏, materials ❏, processes ❏ 2. Method(s) to ensure/enhance quality of measurement/instrumentation ❏  3. Manage non-participation ❏, withdrawal ❏, incomplete/lost data ❏ | | | |  | | | |
|  | | **Is it worth continuing?** | | | | **Data collection [/5]** | | | |  | |
| 6. Ethical matters | | | | | | | | | | | |
| Participant ethics | | | | 1. Informed consent ❏, equity ❏  2. Privacy ❏, confidentiality/anonymity ❏ | | | |  | | | |
| Researcher ethics | | | | 1. Ethical approval ❏, funding ❏, conflict(s) of interest ❏  2. Subjectivities ❏, relationship(s) with participants/cases ❏ | | | |  | | | |
|  | | **Is it worth continuing?** | | | | **Ethical matters [/5]** | | | |  | |
| 7. Results | | | | | | | | | | | |
| Analysis, Integration, Interpretation method | | | | 1. A.I.I. method(s) for primary outcome(s)/output(s)/predictor(s) chosen ❏ and why ❏ 2. Additional AII methods (e.g. subgroup analysis) chosen ❏ and why ❏  3. Suitability of analysis/integration/interpretation method(s) ❏ | | | |  | | | |
| Essential analysis | | | | 1. Flow of participants/cases/groups through each stage of research ❏  2. Demographic and other characteristics of participants/cases/groups ❏  3. Analyse raw data ❏, response rate ❏, non-participation/withdrawal/incomplete/lost data ❏ | | | |  | | | |
| Outcome, Output,  Predictor analysis | | | | 1. Summary of results ❏ and precision ❏ for each outcome/output/predictor/measure 2. Consideration of benefits/harms ❏, unexpected results ❏, problems/failures ❏  3. Description of outlying data (e.g. diverse cases, adverse effects, minor themes) ❏ | | | |  | | | |
|  | |  | | | | **Results [/5]** | | | |  | |
| 8. Discussion | | | | | | | | | | | |
| Interpretation | | | | 1. Interpretation of results in the context of current evidence ❏ and objectives ❏ 2. Draw inferences consistent with the strength of the data ❏  3. Consideration of alternative explanations for observed results ❏  4. Account for bias ❏, confounding/effect modifiers/interactions/imprecision ❏ | | | |  | | | |
| Generalisation | | | | 1. Consideration of overall practical usefulness of the study ❏  2. Description of generalisability (external validity) of the study ❏ | | | |  | | | |
| Concluding remarks | | | | 1. Highlight study's particular strengths ❏  2. Suggest steps that may improve future results (e.g. limitations) ❏  3. Suggest further studies ❏ | | | |  | | | |
|  | |  | | | | **Discussion [/5]** | | | |  | |
| 9. Total | | | | | | | | | | | |
| Total score | | | | 1. Add all scores for categories 1–8 | | | |  | | | |

**Note.** Scoring for each category is based on the guiding principles recommended in the Crowe Critical. Crowe Critical Appraisal Tool (CCAT): Version 1.4 (November 19 2013): Michael Crowe (michael.crowe@my.jcu.edu.au).
