# Supplementary material for: A scoping review of EFL learners’ interlanguage pragmatic development
Source: PLoS One. 2026 Mar 13;21(3):e0344811. doi: 10.1371/journal.pone.0344811 (PMC12987429; doi:10.1371/journal.pone.0344811)
Supplement: S2 Table — (DOCX) [file pone.0344811.s002.docx]

S2 Table. Data extraction and charting of 56 articles

| No. | Authors (Year)  Country | Title | Objective(s) | Study design | Participants | Measures/  Instruments | Main Findings | CCAT Scores |
| --- | --- | --- | --- | --- | --- | --- | --- | --- |
| 1 | Savić et al. (2021) [12]  Norway | Young Greek Cypriot and Norwegian EFL learners: Pragmalinguistic development in request production | Examine request development in Greek Cypriot and Norwegian English learners. | Cross-sectional | 88 Greek, 79 Norwegian EFL learners | Request production tasks | In Norwegian students, there is a clear developmental path. Age-related differences in request strategies, modal verbs, lexical downgrading, and supportive moves were found. | 36 |
| 2 | Eslami et al. (2022) [15]  United States | Variation Patterns in Interlanguage Pragmatics: Apology Speech Act of EFL Learners vs. American Native Speakers | Examine the relationship between pragmalinguistic and sociopragmatic competence among Iranian EFL learners at different proficiency levels. | Cross-sectional | Iranian EFL learners (varied proficiency) | WDCT (apology scenarios) | Across language proficiency levels, both sociopragmatic and pragmalinguistic development was observed. | 40 |
| 3 | Schneider (2021) [17]  Germany | Notes on variational metapragmatics | Investigate variational metapragmatics, with a focus on meta-illocutionary lexicon. | Exploratory corpus-based | English speakers in ICE corpora | ICE-Ireland and ICE-Canada corpora | There are differences in the use of meta-illocutionary expressions in interaction between ICE-Ireland and ICE-Canada. | 37 |
| 4 | Saleem and Saleem (2023) [27]  Pakistan | Pragmatic transfer in congratulation strategies of Punjabi EFL learners: Social power in focus | Investigate the preferences for congratulatory speech acts in specific social situations among Punjabi EFL learners from three social status groups. | Cross-sectional | Punjabi EFL learners (Pakistan) | Elicited congratulation speech acts | The findings show a significant difference between Punjabi EFL learners and British English speakers in their use of congratulatory strategies. | 38 |
| 5 | Napoli and Tantucci (2022) [28]  Italy | Pragmalinguistic and sociopragmatic patterns of requestive acts in English and Italian: Insights from film conversation | Examine the effect of (in)directness and social distance on illocutionary modification of requestive speech acts in dialogic filmic interaction. | Corpus-based | English and Italian speakers (films) | Film dialogue corpus (requests) | The use of mitigating vs. intensifying strategies is significantly influenced by social distance and (in)directness. | 38 |
| 6 | Myrset (2022) [35]  Norway | 'You could win Masterchef with this soup. Can I get some more?' Request production and the impact of instruction on young EFL learners | Investigate the effect of concept-based instruction on requests among young English learners (aged 12-13) in a Norwegian primary school. | Instructional quasi-experimental | Two intact classes of young EFL learners | VODCT (video oral DCT) | After instruction, learners' request production showed increased variation, including the use of modal verbs and supportive moves. | 36 |
| 7 | Kim and Michel (2023) [36]  United States | Linguistic alignment in second language acquisition: A methodological review | Explore the methodological aspects of L2 alignment research | Methodological review | Studies from 2001–2021 (no direct human sample) | Published alignment studies | Syntactic alignment in L2 English is the most widely researched. | 38 |
| 8 | Almusallam (2023) [43]  Saudi Arabia | Offers in Saudi EFL talks: A focus on the learners' pragmatic competence in interactions | Investigate the learners' interactional tools and potential pragmatic transfer. | Naturalistic comparative | 9 Saudi EFL learners + Saudi Arabic and British English NS | Recorded offer interactions | Saudi EFL learners use limited negotiation of offers | 38 |
| 9 | Economidou-Kogetsidis (2023) [44]  Cyprus | "The language is very formal and appropriate": L2 learners' in/appropriateness evaluations and metapragmatic judgments in student-faculty emails | Examine the pragmatic judgments and metapragmatic explanations of Greek EFL learners in relation to student-faculty emails. | Mixed-method | Greek EFL learners | Email ratings + metapragmatic comments | In evaluating status-incongruent emails, low sociopragmatic and pragmalinguistic awareness was observed. | 36 |
| 10 | Littlemore and Fielden-Burns (2023) [45]  United Kingdom | On the fringes of metaphor: Using ambiguously figurative vague language to pragmatically negotiate sensitive topics in the English as a Medium of Instruction classroom | Examine how these three key elements interact: figurative language (specifically metaphor and metonymy), vague language, and sensitive topics. | Qualitative discourse analysis | Two classes of EMI students | EMI classroom recordings | L2 users use the ambiguity inherent in figurative language to discuss complex topics in a pragmatically sensitive manner. | 37 |
| 11 | Dippold (2023) [46]  United Kingdom | "Can I have the scan on Tuesday?" User repair in interaction with a task-oriented chatbot and the question of communication skills for AI | Investigate user repair strategies in interactions with a task-oriented chatbot for scheduling health appointments. | Qualitative | Simulated users scheduling health appointments | User–chatbot interaction transcripts | Repair strategies that are frequently used (for example, rephrasing) may not prompt the bot to correctly recognise intent. Intent recognition is more successful with less commonly used self-repair strategies (e.g., restating the intent). | 38 |
| 12 | Haselow (2021) [47]  Germany | The acquisition of pragmatic markers in the foreign language classroom: An experimental study on the effects of implicit and explicit learning | Investigate the use of pragmatic markers (PMs) by adolescent German-speaking L2 English learners in the foreign language classroom. | Classroom experiment | 18 German L2 English learners | Speaking tasks coded for PMs | Explicit teaching had a short-term effect, increasing the frequency and diversity of PM usage in conversational L2 speech. | 37 |
| 13 | Chen and Lin (2021) [48]  Taiwan, China | Effects of peer collaboration on EFL learners' comprehension of conversational implicatures | Examine the effects of peer collaboration on EFL learners' understanding of conversational implicatures in individual vs. collaborative work | Comparative | 33 Taiwanese EFL learners | MDCT (implicature scenarios) | Negotiation and scaffolding were aided by pair collaboration. | 36 |
| 14 | Zhang (2021) [49]  United States | Combining computer-mediated communication with data-driven instruction: EFL learners' pragmatic development of compliment responses | Investigate the impact of CMC with data-driven instruction on the pragmatic development of EFL learners. | Mixed-method quasi-experimental | 59 Chinese university EFL students | CMC tasks + data-driven instruction | CMC, combined with data-driven instruction, improved the experimental group's appropriateness and variety of CR. | 37 |
| 15 | Derakhshan and Bai (2025) [50]  Iran | Postgraduate Chinese EFL learners' emotional vulnerability displays and regulation strategies | Explore Chinese postgraduate EFL students' emotional vulnerabilities and the strategies they use to regulate these emotions in second language learning. | Qualitative interview study | 20 Chinese postgraduate EFL students | Semi-structured interviews | Students exhibited emotional vulnerabilities through negative responses, facial expressions, suppression, help-seeking, and avoidance, and used both antecedent-focused and response-focused strategies to regulate these experiences. | 37 |
| 16 | House et al. (2021) [51]  Germany | Altered speech act indication: A problem for foreign language learners? | Investigate how Chinese EFL learners evaluate altered speech-act indicators of conventionalized expressions. | Corpus-based | Chinese learners of English | Altered speech-act indication tasks/corpus | All types of altered speech act indications present problems for Chinese learners of English | 36 |
| 17 | Glaser and Martínez-Flor (2025) [52]  Germany | Tracing a pre-service primary English teacher's development in teaching L2 pragmatics | Examine knowledge, beliefs, and challenges of a pre-service teacher during pragmatics instruction in a primary EFL context. | Design-based research | 1 German pre-service teacher; two Grade-4 classes | Class observations + interviews + reflections | The teacher showed gains in pedagogical content knowledge, confidence, and more positive attitudes toward L2 pragmatics teaching; highlighted the need for practical models and improved instruction. | 35 |
| 18 | Wang et al. (2024) [53]  China | Exploring Chinese and Iranian EAP students' oral communication apprehension in English | Examine levels, causes, and coping strategies of oral communication apprehension (OCA) among Chinese and Iranian EAP students. | Cross-cultural mixed-methods | 1618 EAP students (China: 1015; Iran: 603) | OCA questionnaires + semi-structured interviews | Chinese students had higher overall OCA; cultural differences influenced causes and coping strategies; both groups highlighted instructional and psychological factors. | 37 |
| 19 | Zhu and Wang (2022) [56]  United States | Disagreement by Chinese speakers of English: evidence of pragmatic transfer | Investigate pragmatic transfer in Chinese English speakers in disagreement. | Naturalistic conversation analysis | Chinese speakers of English | Conversation corpora (disagreements) | L1/L2 English disagreement differs significantly. | 36 |
| 20 | Suwignyo et al. (2024) [58]  Indonesia | Interlanguage impoliteness in criticism by Javanese-background English learners | Examine the use of impoliteness across social status and distance among Javanese-background English learners. | Mixed-methods role-play study | 40 Indonesian EFL learners | Role-play tasks (criticism) | Negative impoliteness dominated; social status affected impoliteness in familiar but not unfamiliar relationships; high intonation linked to impoliteness. | 36 |
| 21 | Su and Chen (2024) [59]  United States | Examining refusal and acceptance sequences employing a data-driven binary rating approach | Develop a data-driven binary rating rubric to assess roleplay-elicited pragmatic performance in L2 Chinese, focusing on refusal and acceptance sequences. | Analytic discourse-based | 54 L2 Chinese learners + 22 native speakers | Role-play tasks with binary rating rubric | Lower-proficiency learners struggled more with refusing return-for-favor intentions; higher proficiency was associated with better sequential organization. The rubric showed high reliability and practicality. | 37 |
| 22 | Xiao et al. (2021) [60]  China | The development of interlanguage pragmatic markers in alignment with role relationships | Examine the dynamic development of interlanguage pragmatic markers (PMs) in EFL contexts. | Longitudinal | 28 EFL learners | Pragmatic marker use over time | Students with varying levels of proficiency experienced different levels of PM development. | 35 |
| 23 | Pan (2023) [61]  Thailand | Formulaic Language Use by Learners of English in Interlanguage Communication | Investigate the use of four-word lexical bundles in interlanguage communication by Thai English learners at various proficiency levels. | Observational | 120 Thai ESL learners (three proficiency levels) | Spoken interlanguage corpus | Higher English proficiency EFL learners use less formulaic language. | 36 |
| 24 | Pouromid and Hosseininasab (2022) [62]  Japan | Demonstrating active listenership through collaborative turn completion to display epistemic access in multi-party interactions | Investigate how active listening affects co-participation in interaction. | Conversation analysis (case study) | Students in a linguistics course (Japan) | Classroom interaction recordings | Active listening to demonstrate epistemic access is a subtle act of participation that can significantly impact how participants construct a course of action. | 37 |
| 25 | Hendriks et al. (2023) [63]  Netherlands | The effects of L1 and L2 writers' varying politeness modification in English emails on L1 and L2 readers | Examine the differences in politeness evaluation between L1 and L2 English email requests. Modifications. | 2×2 experiment | Dutch and British participants | Email-request rating tasks | Less politeness in L2 emails led to perceptions of bossiness, greater authority, and a greater willingness to comply. | 35 |
| 26 | Alshraah et al. (2024) [64]  Saudi Arabia | Pragmatic Realization in Exploiting Request Expressions: A Study of Language Proficiency and Social Context among Saudi EFL Learners | Investigate the impact of language proficiency and social context on Saudi EFL learners' pragmatic realization of request strategies. | Cross-sectional | 98 first-year Saudi EFL students (50 high, 48 low achievers) | DCT + SRQ (requests) | High achievers showed significantly greater pragmatic competence and a greater use of mitigated and polite request strategies than low achievers. Language proficiency is positively linked to better pragmatic realization. | 35 |
| 27 | Siddiqa and Whyte (2025) [67]  Switzerland | Classroom learning of English L2 requests: Input and interactional opportunities in French secondary schools. | Investigate how textbooks, classroom interaction, and teacher beliefs shape French EFL learners' opportunities to acquire L2 requests. | Cross-sectional mixed-methods | 308 French secondary EFL students; 10 EFL teachers | Textbook analysis + classroom observation + teacher interviews | Textbooks and classroom input offered limited pragmatic support; teacher beliefs and practices influenced learners' opportunities to make L2 requests; pragmatic development opportunities were minimal across levels. | 36 |
| 28 | Soleimani et al. (2021) [68]  Iran | Feasibility of Using a Task-Oriented Focus on Form Instructional Model for the Study of Request Speech Act | Examine the effect of form-focused instruction on instructed interlanguage pragmatics (IILP). | Classroom experiment | 90 university students | Request performance pre-/post-tests | The ability of learners in the instructed group to perform the request speech act improved. | 37 |
| 29 | Nergis (2021) [69]  Turkey | Can explicit instruction of formulaic sequences enhance L2 oral fluency? | Examine the effects of explicit learning of formulaic sequences on advanced EAP learners' L2 utterance fluency. | Experimental | Two groups of first-year students | Oral fluency speaking tasks | For EFL learners, explicit instruction and practice of formulaic language in language instruction are beneficial. | 36 |
| 30 | Luo and Derakhshan (2024) [70]  China | Examining the role of classroom climate, teacher-student relationships, and EFL students' perceived learning outcomes | Explore the associations and predictive roles of classroom climate and teacher-student relationship on Chinese EFL students' perceived learning outcomes. | Quantitative survey (SEM) | 506 Chinese EFL students (ages 16–22+) | Questionnaires (classroom climate, TSR, outcomes) | A favorable classroom climate and teacher-student relationships jointly accounted for 39.8% of perceived learning outcomes; both factors significantly predicted these outcomes. | 38 |
| 31 | Rafiq and Yavuz (2024) [71]  Turkey | Advancing English as second language communication: The effectiveness of online pragmatic training on essential speech acts | Investigate if online implicit/explicit pragmatic training improves ESL learners' pragmatic competence. | Randomized experiment | 60 Pakistani university students | Online pragmatic training + DCT pre-/post-tests | Both training types improved pragmatic competence significantly vs. control; the explicit group showed a larger effect size, though no significant difference between groups. | 37 |
| 32 | Khazaie & Derakhshan (2024) [72]  Iran | Extending embodied cognition through robots' augmented reality in English for medical purposes classrooms | Investigate the effect of robots' augmented reality on Saudi EFL learners' embodied cognition and comprehension in English for Medical Purposes classrooms. | Quasi-experimental (flipped classroom) | 526 Saudi EFL students | Robot-AR medical English lessons + comprehension tests | Students using the robot's AR achieved significantly higher comprehension scores in English for Medical Purposes listening and reading tasks than the control and robot-only groups | 36 |
| 33 | Yan Cong (2024) [73]  United States | Demystifying large language models in second language development research | Explore LLM-Surprisal as an index for L2 English writing development and assessment. | Quantitative computational | 297 Chinese L2 English learners | Learner writing corpora + LLM surprisal indices | LLM-Surprisal effectively detected L2 writing, indexed proficiency stages, and supplemented classic NLP indices; Surprisal reflects the interplay between lexical diversity and syntactic complexity in L2 development. | 36 |
| 34 | Al-Rawafi et al. (2021) [75]  Indonesia | Students' apologizing in Arabic and English: An interlanguage pragmatic case study at an Islamic boarding school in Indonesia | Investigate the impact of external versus internal contextual factors on students' apologising and pragmatic transfer. | Cross-sectional | 101 male, 101 female students | Written DCT (apologies) | Students demonstrated on-record-negative politeness attitudes influenced by contextual factors and a lack of grammatical competence. | 35 |
| 35 | Al-Harbi and Mahfoodh (2021)  [76]  Malaysia | The production and comprehension of apology strategies: Effects of English language proficiency | Determine how English proficiency level shapes Jordanian EFL students' ability to produce and comprehend apology strategies. | Cross-sectional comparative | 270 Jordanian EFL learners + 90 native-English speakers | Written DCT + MDCT (apologies) | Higher‑proficiency learners used fewer explicit or non‑apology forms, favored subtler apologies, and understood apology strategies better. | 37 |
| 36 | Köylü et al. (2023) [80]  Switzerland | A dynamic usage-based analysis of L2 written complexity development of sojourners | Examine the relationship between time, holistic judgments of proficiency, and the development of syntactic/lexical complexity. | Longitudinal content analysis | Advanced L2 sojourners abroad | Sojourners’ written texts | The results confirm the significance of random individual variation | 35 |
| 37 | Hao et al. (2021) [81]  China | Typological characteristics of interlanguage: Across native language types and L2 proficiency levels | Examine how interlanguage typology develops among Chinese learners with English and Japanese L1 backgrounds. | Quantitative corpus-based | Learners with L1 English/Japanese | Tagged Chinese interlanguage corpus | Interlanguage shows gradual development influenced by L1 typology and markedness, with shared SV and VO preferences, and dependency direction serving as a universal metric. | 38 |
| 38 | Li (2022) [82]  China | Corpus-Based Error Analysis of Chinese Learners' Use of High-Frequency Verb Take | Compare error patterns of TAKE between two proficiency levels of Chinese learners (ST4 vs. ST6 in CLEC). | Corpus-based CIA + EA | Chinese learners in CLEC ST4 and ST6 subcorpora | CLEC subcorpora ST4 and ST6 (verb take) | ST6 learners err less than ST4 learners, but in both corpora, most TAKE mistakes are wrong-word or collocation errors rooted in L1 transfer and overgeneralization. | 37 |
| 39 | Sögüt and Keçik, (2023) [83]  Turkey | EFL learners' production of verb complementation patterns and verb senses: an investigation on high-frequency cognitive verbs | Examine how Turkish L2 learners deploy the high-frequency cognitive verbs think and believe with respect to (a) verb senses and (b) complementation patterns. | Experimental | 182 EFL students (four vocabulary-proficiency bands) | Sentence-production and sentence-completion tasks (think/believe) | Learners with higher vocabulary levels performed better in both the context-free and context-dependent tasks, with performance becoming more similar across tasks as vocabulary level increased. | 37 |
| 40 | Worathumrong (2021) [84]  Thailand | "How the Furby Coming is…": Interference of First Language and Culture in Thai EFL Learners' Paragraph Writing | Examine how first-language and cultural interference appear in Thai undergraduates' English paragraphs on pop-culture topics, comparing high-exposure (TEH) and low-exposure (TEL) learners. | Mixed-methods text analysis | 30 Thai undergraduates (15 TEH, 15 TEL) | English paragraphs on pop-culture topics (TEH vs TEL) | Both groups used the same metadiscourse and SP/PP/EPP flows, showing an oral, reader-responsible style; high-exposure writers were clearer and less L1-influenced, underscoring the need to teach interference awareness. | 35 |
| 41 | Alsmari (2024) [85]  Saudi Arabia | Learner Autonomy and Interlanguage Pragmatic Learning Strategies (IPLS) Use: A Gender-based Analysis in the Saudi EFL Context | Examine the relationship between IPLS use and learner autonomy among male and female Saudi EFL learners, and investigate gender-based differences. | Quantitative correlational | 156 Saudi upper-intermediate EFL learners (78 males, 78 females) | IPLS/autonomy surveys + proficiency tests | Female learners showed higher autonomy and more strategic IPLS use. A strong correlation between IPLS and autonomy was observed in males and a moderate one in females. | 38 |
| 42 | Carmen Pérez-Llantada (2024) [87] Spain | Approaching digital genre composing through reflective pedagogical praxis | Critically reflect on teaching digital genre composing for professional and public science communication. | Exploratory case study | 12 STEM researchers (University of Zaragoza) | Digital genre texts + reflective writings | Raising students' genre awareness helped in composing digital genres. Students could recontextualize scientific content, but struggled to simplify language for broader audiences. Explicit instruction in metageneric strategies is recommended. | 38 |
| 43 | Lee et al. (2023) [89]  United States | Navigating the Interlanguage Space: Chinese International Students' Perceptions of a Virtual Chemistry Laboratory Course | To explore how Chinese EFL learners perceived and navigated linguistic challenges in a virtual chemistry lab course during COVID-19. | Qualitative case study | Chinese international students in a US university | Interviews on virtual chemistry lab course | Students found the virtual lab low-pressure but struggled with understanding the procedures. Strategies used mainly supported surface learning; deeper engagement with academic language was limited. | 36 |
| 44 | Schauer (2022) [92]  Germany | Exploring the potential of graphic novels for L2 pragmatic teaching and learning–focus on young learners | Investigate the potential of graphic novels to provide practical support for young L2 learners at the beginner and intermediate levels. | Content analysis | N/A (textual data) | Four graphic novels (speech acts) | The research provided an overview of the frequency of eight speech acts, along with their expressions and formulaic routines. | 36 |
| 45 | Almalki and Jones, (2022)  [93]  United Kingdom | 'Why did you do that?' The effects of instruction on recognition and production of informal second-party complaints | Examine whether targeted instruction improves (1) learners' receptive knowledge and (2) productive appropriacy of informal second-party complaints exchanged among peers. | Quasi-experimental pre-/post-test | 30 Saudi male undergraduate EFL students | Complaint recognition + production tests | Instruction led to a significant gain in receptive complaint recognition, but no significant improvement in the appropriacy of produced complaints. | 36 |
| 46 | Xu et al. (2024) [94]  UK | Task-based pronunciation teaching: Lack of auditory precision, but not memory, hinders learning | Examine how individual differences in auditory processing and working memory affect the Effectiveness of task-based pronunciation teaching (TBPT) on vowel perception among Chinese EFL learners. | Quasi-experimental | 70 Chinese EFL learners (50 experimental, 20 control) | Forced-choice vowel identification tests | TBPT led to significant improvements in vowel perception (~10% gains); auditory processing (AP) significantly mediated learning outcomes, but working memory (WM) did not; learners with normative AP benefited more than those with low AP. | 37 |
| 47 | Bardovi-Harlig et al. (2022) [S1]  United States | Recognition of conventional expressions by EFL learners in Mexico and China | Investigate the role of EFL instructional contexts in the acquisition of pragmalinguistic resources such as conventional expressions. | Comparative cross-sectional | 303 EFL learners + 89 native speakers (US) | Conventional-expression recognition test | Instruction and media access were identified as variables influencing pragmatic development in EFL contexts. | 38 |
| 48 | Hosseinpur and Kazemi (2022) [S2]  Iran | Composing strategies employed by high-and low-performing Iranian EFL students in essay writing classes | Within the context of L2 writing research, investigate the use of writing strategies in various sociocultural contexts. | Descriptive | 58 Iranian EFL writers | Essays + composing-strategy data | Metacognitive strategies significantly predict English writing ability | 35 |
| 49 | Su et al. (2022) [S3]  China | Assessing pragmatic performance in advanced L2 academic writing through the lens of local grammars: A case study of 'exemplification' | Investigate the relationship between L2 English writing proficiency and pragmatic exemplification performance. | Case study | Chinese undergraduate and postgraduate EFL learners + expert writers | Academic writing corpora (exemplification) | Proficiency is associated with increased frequency, quantity, strategic use, and variety of exemplificatory markers and local grammar patterns. | 35 |
| 50 | Xiao et al. (2023) [S4]  China | A multidimensional alignment sustainability model for language development: Evidence from L1 and L2 semio-semantic and semio-pragmatic markers | Investigate language development within the Ecolinguistic Continuum. | Corpus-based | Chinese L1 and English L2 corpora | L1/L2 marker distributions | There are differences in marker use distributions between L1 and L2, indicating multidimensional niches for long-term alignment development. | 37 |
| 51 | Wu and Roever (2025) [S5]  Germany | Data from role plays and elicited conversations: L2 interactional competence | Compare role plays and elicited conversations in capturing features of L2 interactional competence. | Mixed-methods comparative | 54 L2 Chinese learners; 12 Chinese L1 speakers | Role plays + elicited conversations | Role plays better capture dispreferred actions (e.g., requests, refusals); elicited conversations better capture topic management; both methods are complementary but non-interchangeable. | 36 |
| 52 | Derakhshan et al. (2024)  [S6]  Iran | Is game-based language learning general or specific? Exploring the applicability of mobile virtual realities to medical English education in the Middle East | Investigate whether mobile VR-based educational games enhance general English and English for Medical and Academic Purposes (EMAP) productive skills among medical students in Middle Eastern universities. | Quasi-experimental parallel groups | 673 general English and 522 EMAP students | VR vs traditional EMAP speaking/writing tests | Mobile VRs significantly improved EMAP speaking and writing skills more than general English skills; they provided more authentic, lifelike environments for learning medical English communication than traditional online modules. | 37 |
| 53 | Behzad Pourgharib and Farzaneh Shakki (2024) [S7]  Iran | The interplay between English teachers' rapport and immediacy and the students' academic motivation | Investigate how English teachers' rapport and immediacy behaviors affect EFL students' academic motivation, and whether gender differences exist. | Quantitative SEM study | 504 Iranian EFL learners (18–23 years) | Rapport, immediacy, and motivation questionnaires | Teachers' rapport/immediacy strongly predicted students' motivation (57% variance explained); gender effects were negligible. | 36 |
| 54 | Tajeddin and Bagherkazemi (2021) [S8]  Iran | Implicit and Explicit Pragmatic Learning Strategies: Their Factorial Structure and Relationship with Speech Act Knowledge | Create a pragmatic learning strategy inventory (PRALSI) and investigate its relationship with knowledge of speech acts. | Mixed-method | 245 Iranian English-major undergraduates | PRALSI questionnaire + speech-act knowledge tests | The findings shed light on the implications of the implicit/explicit learning dichotomy for pragmatic development and strategy use, as well as the potentially greater advantage explicit strategies may provide for pragmatic knowledge. | 37 |
| 55 | Yuliani et al. (2023)  [S9]  Indonesia | Engaging Indonesian students in the "read, reread, list, compose" strategy to enhance paraphrasing skills. | Explore the Effectiveness of the Read, Reread, List, Compose (RRLC) strategy in enhancing university students' paraphrasing skills in an Indonesian college context. | Qualitative case study | 9th-semester university students in Bandung | Classroom observations + paraphrasing tasks (RRLC) | The RRLC strategy enhanced students' paraphrasing skills by improving text comprehension, encouraging structural variation, and promoting the use of synonyms while preserving original meaning. | 37 |
| 56 | Carrió-Pastor (2021) [S10]  Spain | The assessment of metadiscourse devices in English as a foreign language | Identify metadiscourse devices in essays written by English learners at various CEFR levels. | Corpus-based | English learners (varied CEFR levels) | Learner essay corpus | Metadiscourse devices were classified according to their proficiency level. | 35 |
| Note. WDCT = written discourse completion task; DCT = discourse completion task; MDCT = multiple-choice discourse completion task; OCA = oral communication apprehension; SEM = structural equation modeling; EMAP = English for medical and academic purposes; VR = virtual reality; CMC = computer-mediated communication; EMI = English as a medium of instruction; TSR = teacher–student relationship; CIA = contrastive interlanguage analysis; EA = error analysis; RRLC = read, reread, list, compose; TEH = high-exposure group; TEL = low-exposure group.  S2 References  S1. Bardovi-Harlig K, Izquierdo J, Su Y. Recognition of conventional expressions by EFL learners in Mexico and China. System. 2022;110: 102918. doi:10.1016/j.system.2022.102918  S2. Hosseinpur RM, Kazemi Z. Composing strategies employed by high-and low-performing Iranian EFL students in essay writing classes. Assessing Writing. 2022;51: 100601. doi:10.1016/j.asw.2021.100601  S3. Su H, Lu X. Assessing pragmatic performance in advanced L2 academic writing through the lens of local grammars: A case study of ‘exemplification.’ Assessing Writing. 2022;54: 1–12. doi:10.1016/j.asw.2022.100668  S4. Xiao HZ, Zhang W, Mo R. A multidimensional alignment sustainability model for language development: Evidence from L1 and L2 semio-semantic and semio-pragmatic markers. Language Sciences. 2023;100: 101572. doi:10.1016/j.langsci.2023.101572  S5. Wu J, Roever C. Data from role plays and elicited conversations: What do they show about L2 interactional competence? Research Methods in Applied Linguistics. 2025;4: 100165. doi:10.1016/j.rmal.2024.100165  S6. Derakhshan A, Teo T, Khazaie S. Is game-based language learning general or specific-oriented? Exploring the applicability of mobile virtual realities to medical English education in the middle east. Computers & Education. 2024;213: 105013. doi:10.1016/j.compedu.2024.105013  S7. Pourgharib B, Shakki F. The interplay between English teachers’ rapport and immediacy and the students’ academic motivation. Learning and Motivation. 2024;87: 101991. doi:10.1016/j.lmot.2024.101991  S8. Tajeddin Z, Bagherkazemi M. Implicit and explicit pragmatic learning strategies: Their factorial structure and relationship with speech act knowledge. Tesl-EJ. 2021;25: 1–28. Available: https://www.tesl-ej.org/pdf/ej99/a5.pdf  S9. Yuliani T, Kurniawati N, Siswayani P. Engaging Indonesian students in “read, reread, list, compose” strategy to enhance paraphrasing skill. Journal of Education and Learning. 2023;17: 195–205. doi:10.11591/edulearn.v17i2.20438  S10. Carrió-Pastor ML. The assessment of metadiscourse devices in English as a foreign language. Assessing Writing. 2021;50: 100560. doi:10.1016/j.asw.2021.100560 | | | | | | | | |
